# Prevalence of selected infectious agents in Swedish cats with fever and/or anemia compared to cats without fever and/or anemia and to stable/stray cats

**DOI:** 10.1186/s13028-025-00807-8

**Published:** 2025-05-09

**Authors:** Gunilla Ölmedal, Linda Toresson, Mary Nehring, Jennifer Hawley, Sue Vande Woude, Michael Lappin

**Affiliations:** 1 Evidensia Specialist Animal Hospital, Bergavägen 3, Helsingborg, 25466 Sweden; 2 Department of Equine and Small Animal Medicine, Faculty of Veterinary Medicine, Helsinki University, Agnes Sjobergin katu 2, Helsinki, 00014 Finland; 3 Department of Microbiology, Immunology, and Pathology, College of Veterinary Medicine and Biomedical Sciences, Colorado State University, Fort Collins, CO USA; 4 Center for Companion Animal Studies, Department of Clinical Sciences, College of Veterinary Medicine and Biomedical Sciences, Colorado State University, 300 West Drake Road, Fort Collins, CO 80523 USA

**Keywords:** *Bartonella*, *Ehrlichia*, Feline, Feline foamy virus, Feline immunodeficiency virus, Feline leukemia virus, Felis catus gammaherpesvirus 1, *Mycoplasma*, *Toxoplasma gondii*

## Abstract

**Background:**

There are multiple infectious agents of cats around the world; those transmitted by direct contact among cats, hunting, or exposure to fleas or ticks are frequently the most common. Some infectious disease agents have been reported in cats in Sweden; for example, *Anaplasma phagocytophilum* infection was first reported in a cat in this country. However, there has not been a study in Sweden that reported test results for agents with different transmission cycles in cats with and without signs of clinical disease. Thus, the aims of this study were to (i) investigate prevalence of exposure to *Anaplasma* species, *Bartonella* species, *Ehrlichia* species, haemotropic *Mycoplasma* species, feline foamy virus (FFV), Felis catus gammaherpesvirus (FcaGHV1), feline immunodeficiency virus (FIV), feline leukemia virus (FeLV) and *Toxoplasma gondii* in cats residing in the Southern part of Sweden (ii) compare prevalence in samples between 3 groups of cats (cats with fever and/or anemia, cats without any signs of infectious disease, and cats that were either stray cats or stable cats).

**Results:**

Overall, antibodies were detected against FcaGHV1 (67%, CI 57–76%), FFV (45%, CI 35–55%), *Bartonella* species (43%, CI 34–54%), *T. gondii* (37%. CI 28–47%), and FIV (3.3%, CI 1.1–9.2%). FeLV antigen was detected in one cat (1.1%, CI 0.19–5.9%). Haemotropic *Mycoplasma* DNA was amplified in seven cats (7.6%, CI 3.7–15%). All five samples with successful sequencing were ‘*Candidatus* M. haemominutum’. The one cat (1.1%, CI 0.19–5.9%) that was positive for *B. henselae* DNA also had a *Bartonella* spp. titer of 1:1024. *Anaplasma* and *Ehrlichia* spp. DNA were not amplified from any cat.

**Conclusions:**

The antibody test results suggest that many of these cats were exposed to other cats (FFV, FcaGHV1, FIV, FeLV), had inadequate flea control (*Bartonella* spp.), and were fed undercooked meat or allowed to hunt (*T. gondii).* While infection was common, the only haemotropic *Mycoplasma* amplified from these cats was the relatively non-pathogenic ‘*Candidatus* M. haemominutum*’*. As previously documented for each of these agents, the presence of a positive test result or infection by one or more organisms is not always associated with illness.

**Supplementary Information:**

The online version contains supplementary material available at 10.1186/s13028-025-00807-8.

## Background

As a result of the behavioral characteristics and housing of cats, infectious agents transmitted by exposure to fleas or ticks, direct contact among cats, or hunting are frequently the most common. Most of the agents can be associated with clinical illness like fever and anemia and some are zoonotic [1]. Thus, it is important to investigate the prevalence of common feline infectious disease agents in all regions of the world.

The most common tick transmitted agents associated with clinical illness in cats have been *Anaplasma phagocytophilum*, *A. platys*, *Borrelia burgdorferi*, *Cytauxzoon felis*, and *E. canis* [2]. The most common flea associated agents include *Bartonella henselae*, *B. koehlerae*, *B. clarridgeiae*,* Rickettsia felis*, and potentially, haemotropic *Mycoplasma* spp [3]. The distribution of these agents generally mirrors the distribution of the vectors. When clinical signs from a flea or tick associated disease occurs in cats, fever is generally most common [2, 3]. Sweden is endemic for *Ctenocephalides felis* and *Ixodes* spp., thus, *A. phagocytophilum* and some *Bartonella* spp. are known to occur. The first case report of *A. phagocytophilum* in a cat was from Sweden [4]. Since the first report, this agent has been detected in several cats with fever, lethargy and anorexia [4–7] in a number of studies around the world, but experimental infections have not induced measurable signs of disease [8]. *Anaplasma phagocytophilum* is endemic in multiple species in Sweden [9–12] and *A. phagocytophilum* antibodies were detected in 21% of the cat sera tested in central Sweden in 2010–2011 [13]. There is no study reporting the prevalence of DNA from *A. phagocytophilum* in blood of Swedish cats. Antibodies against *A. phagocytophilum* can cross react against *A. platys* in some assays, so results of seroprevalence studies do not differentiate the agents. *Ehrlichia canis* is a monocytotropic agent transmitted by *Rhipicephalus sanguineus* that has been associated with illness, including fever and anemia in some cats [14–16]. This tick is not currently distributed to Sweden [17], likely because the cool climate has prevented establishment of an outdoor breeding population [18]. In addition, there are no published cases of *E. canis* in cats in this country and none have been reported to the Swedish Board of Agriculture (https://www.government.se/government-agencies/swedish-board-of-agriculture/). Thus, this infection would be unlikely unless the cat had traveled from an endemic area like Spain [19, 20]. However, with global climate changes, the distribution of ticks is changing. There is no validated serological test for use with cat sera for *Anaplasma* spp. or *Ehrlichia* spp. Thus, PCR assays are used to amplify DNA of the agents to prove current infection.

The most common flea associated agents in cats are *Bartonella* spp. and *Rickettsia felis* [3, 21]. Both agents are found in flea feces and can be zoonotic [3]. One of the most common signs of bartonellosis in experimentally infected or naturally exposed cats is fever [22–24]. While flea associated *Bartonella* spp. in cats have an intra-erythrocytic stage, anemia has been uncommon [24, 25]. It is likely that the intra-erythrocytic phase is a host evasion mechanism to allow the agent to be ingested by the flea while taking the blood meal. To date, two previous studies have investigated the prevalence of *Bartonella* spp. in Swedish cats [26, 27]. Assays to detect serum antibodies or amplify DNA of the agent are available in some countries.

*Mycoplasma haemofelis*, ‘*Candidatus* M. haemominutum*’* and ‘*Candidatus* M. turicensis’ are haemotropic *Mycoplasma* spp. with varying degrees of virulence. In most studies, *M. haemofelis* has been the most pathogenic species, with fever or hemolytic anemia most common when illness occurs [28–31]. The first description of *Mycoplasma haemofelis* in a cat in Sweden was in 1978 [32]. However, large scale prevalence studies from Sweden are lacking. There is no commercially available serological test for these agents, so PCR assays are used to amplify DNA and confirm current infection. These agents were believed to be flea transmitted but that route has been difficult to prove [33, 34], so now many investigators believe direct or vertical transmission to be most likely [35].

There are 3 exogenous retroviruses known to infect cats [36]: Feline leukemia virus (FeLV), feline immunodeficiency virus (FIV) and feline foamy virus (FFV). These agents are most commonly transmitted among cats by direct exposure. FeLV and FIV are well characterized pathogens, have been detected in cats in Sweden, and are reportable to the Swedish Board of Agriculture [36, 37]. While FFV generally is not associated with clinical disease [38–40], there is a report of FFV proviral load being positively associated with progressive FeLV disease [41]. Tests for serum antigen (FeLV) and antibodies (FIV) are widely available around the world and nucleic acids of all 3 retroviruses can be amplified in molecular assays. FFV antibody assays are available mainly in research laboratories, and to date, prevalence studies on FFV from Scandinavia are lacking.

Felis catus gammaherpesvirus (FcaGHV1) was described for the first time in 2014 and the agent is likely transmitted directly among cats [42]. The majority of positive cats have been healthy and reports whether FcaGHV1 is more common among ill than healthy cats have been contradictory [43–45]. Serum antibody tests are generally only available in research laboratories and the nucleic acids of the agent can be amplified in molecular assays. Felis catus gammaherpesvirus has not been reported from any of the Scandinavian countries and additional studies of coinfection with this agent with other infections are needed.

*Toxoplasma gondii* is a zoonotic parasite that reproduces only in felids [1, 46, 47]. Most cats are infected by carnivorism from hunting or being fed infected, undercooked meat. In a previous Swedish study, a seroprevalence of 42% was reported [48]. After the primary exposure, millions of oocysts are shed into the environment. After sporulation, which occurs in 1–3 days, the oocysts are infectious to all vertebrates. As the definitive host, cats rarely become ill, but clinical cases are described in cats co-infected with FIV or FeLV [49]. Fever is common when signs do occur [50]. While *T. gondii* DNA can be amplified from feces or blood, most prevalence studies generally use serum antibody tests. Once a cat becomes infected, the tissue phase is probably not eliminated.

While there have been a number of infectious agents detected in cats currently living in Sweden, studies reporting test results for agents with different transmission cycles in cats with and without clinical signs of disease are lacking. Thus, the aims of this study were to (i) investigate prevalence of exposure to *Anaplasma* species, *Bartonella* species, *Ehrlichia* species, haemotropic *Mycoplasma* species., feline foamy virus (FFV), Felis catus gammaherpesvirus (FcaGHV1), feline immunodeficiency virus (FIV), feline leukemia virus (FeLV) and *Toxoplasma gondii* in cats residing in the Southern part of Sweden (ii) compare prevalence in samples between 3 groups of cats (cats with fever and/or anemia, cats without clinical signs of infectious disease, and cats that were either stray cats or stable cats).

## Methods

### Study design

Blood samples were collected between 2013 and 2017, with no sampling during January, February or December. All cat owners completed a questionnaire on the background variables of the cat (Additional file 1). Cats treated with antibiotics within 4 weeks prior to inclusion were excluded.

Group 1 consisted of cats with fever and/or anemia that were presented at Evidensia Specialist Animal Hospital in Helsingborg (ESAHH). Inclusion criteria were a rectal temperature < 39.5 ℃ or a hematocrit below 20%. Exclusion criteria were bite wounds or abscesses, ocular or nasal discharge or diarrhea. Cats presenting with vomiting and fever were excluded, but cats with anemia that vomited occasionally during the hospitalization period were not excluded.

Group 2 consisted of control cats without clinical signs of infectious disease. These were typically cats presented for routine surgery, staff-owned healthy cats or cats with either well-characterized chronic illness or trauma, without signs of fever or anemia. Effort was made to match age, indoor/outdoor, sex and time of year it was sampled with cats from group 1, but complete match was not possible.

Group 3 consisted of cats that were either trapped strays, or cats living exclusively outdoors in horse stables or barns in groups exceeding seven individuals. Thirteen of the cats in group 3 had no obvious health problems at clinical examination. Three cats had clinical illnesses (neoplasia, abscess, polyarthritis). It was not possible to match cats in group 3 with the other groups in any aspect.

All samples were collected at ESAHH, in stables by the first author or at the veterinary clinic Kattens Veterinär. Serum and whole blood samples were stored frozen (-18 ℃) and shipped on dry ice to Colorado State University.

### Ethical consent

All cat owners signed an informed written consent. Consent was given either before sampling (in all cases when sampling was done solely for the study, or if increased blood volumes were drawn for the study) or after the visit to the clinic but before the cat was allowed to enter the study (when left over blood samples, drawn for clinical purposes, could be used). The study was approved by the Local Ethics Committee in Malmö Lund (M-398-12) and Uppsala (C78/13), Sweden.

### Serologic testing

Serum samples were tested for FeLV antigen and FIV antibody using a commercially available kit (SNAP Feline Triple Test; IDEXX laboratories, Maine, USA). Previously reported plate-based enzyme-linked immunosorbent assays (ELISA) was used to detect *T. gondii* and *Bartonella*-species specific IgG antibodies using the standard operating procedures in an accredited Veterinary Diagnostic Laboratory (Colorado State University, Fort Collins, CO, USA). FcGHV1 antibodies were measured in sera of the cats using a previously reported assay [51], and antibodies against FFV were detected by ELISA as previously reported [52].

### Polymerase chain reaction assays

Total DNA was extracted from 200 µL of whole blood with QIAcube HT automated purification instrument and QIAamp 96 DNA QIAcube HT kit and QIAamp DNA Blood Mini Kit. Previously described conventional polymerase chain reaction (PCR) assays were used to amplify DNA of *Anaplasma* species and *Ehrlichia* species [5], *Bartonella* species and hemoplasmas [53]. The resulting DNA PCR products were visualized by agarose gel electrophoresis with EZ-Vision One DNA dye (Amresco, Solon, OH, USA). Amplicons from positive PCR assays were sequenced at a core facility (Colorado State University).

### Statistical analysis

Initially associations between the occurrence of agents or antibodies and subject characteristics/background (age, sex, raised indoor/outdoor, access to outdoor, number of other cats in the household, history of tick, fleas, lice and bit wound) was tested. Age was grouped ensuring as even a group size as possible for statistical analysis (Table 1). The chi-square test was applied when feasible (and when appropriate with Yates’ correction for continuity) and otherwise, the Fisher’s test was applied, in its exact form when feasible and otherwise in a simulated version. Post hoc analyses were performed according to the same principles, with the addition of Bonferroni adjustments of P-values. When studying associations between occurrences of agents or antibodies and inclusion group (fever/anemia, control cat or stable/stray), a sequence of analyses analogous to the description above was performed. In order to assess possible confounding in the case of the estimated associations between occurrence of agents and inclusion group, these associations were reassessed using univariate log-binomial models into which the possible confounders (age, sex, raised indoor/outdoor, access to outdoor, number of other cats in the household, history of tick, fleas and bit wound) were subsequently incorporated. The Likelihood Ratio Test (LRT) P-values associated with the inclusion group in these multivariate models were interpreted as adjusted for possible confounding. When studying the cooccurrence of agents (co-infections) the chi-square test with Yates’ correction for continuity was applied when feasible and otherwise, the Fisher’s exact test was applied. No adjustment for multiple testing was applied and no comparison between inclusion groups were made, this being viewed as a purely explorative part of the overall analysis of the data. *P* < 0.05 was considered significant. Prevalence was calculated as number of positive test results divided by total number of samples, without adjustments for sensitivity or specificity of the test method. Confidence intervals were calculated with Sergeant, ESG, 2018. Epitools Epidemiological Calculators. Ausvet, using the “Wilson” method and with confidence level 0.95. All other analyses were performed in R version 3.5.1 using the functions fisher.test(), chisq.test() and glm().

**Table 1:** Background data from the 91 cats in this study (92 samples)

Summary of participating cats	Baseline data at inclusion *n* (%)		
	AF	CC	SS
**Total number**			
**Sex**	42	34	16
Neutered female	14 (33)	16 (47)	6 (38)
Female	1 (2)	1 (3)	3 (19)
Neutered male	27 (64)	13 (38)	5 (31)
Male	0	4 (12)	1 (6)
Unknown	0	0	1 (6)
**Median age (year)**	5	6.5	1
**Age distribution (year) (one cat included twice in AF group)**			
≤ 2	7 (17)	8 (24)	6 (38)
> 2 ≤ 5	10 (24)	7 (21)	4 (25)
> 5 ≤7	17 (40)	6 (18)	0
> 7 ≤ 17	7 (17)	11 (32)	1 (6)
Unknown	1 (2)	2 (6)	5 (31)
**Breed**			
Domestic short- or longhair	27 (64)	21 (62)	8 (50)
Unknown/mixed breed	10 (24)	4 (12)	8 (50)
Norwegian forest cat	1 (2)	4 (12)	0
Ragdoll	1 (2)	2 (6)	0
Siamese	1 (2)	1 (3)	0
Exotic shorthair	0	1 (3)	0
Oriental shorthair	1 (2)	0	0
Siberian	1 (2)	0	0
Birman	0	1 (3)	0
**No of additional cats in household**			
0	17 (40)	9 (26)	0
1	11 (26)	17 (50)	0
2	9 (21)	4 (12)	0
3 or more	3 (7)	2 (6)	14 (88)
Unknown	2 (5)	2 (6)	2 (13)
**Access to outdoor**			
Yes	25 (60)	19 (56)	16 (100)
No	15 (36)	15 (44)	0
Unknown	2 (5)	0	0
**Raised indoor or outdoor**			
Indoor	14 (33)	15 (44)	3 (19)
Outdoor	26 (62)	18 (53)	10 (63)
Unknown	2 (5)	1 (3)	3 (19)
**History of fighting with other cats**			
Yes	19 (45)	9 (26)	14 (88)
No	21 (50)	23 (68)	2 (13)
Unknown	2 (5)	2 (6)	0
**History of bite wound**			
Yes	7 (17)	2 (6)	10 (63)
No	33 (79)	31 (91)	3 (19)
Unknown	2 (5)	1 (3)	3 (19)
**History of fleas**			
Yes	7 (17)	3 (9)	0
No	33 (79)	30 (88)	5 (31)
Unknown	2 (5)	1 (3)	11 (69)
**History of lice**			
Yes	1 (2)	0	0
No	39 (93)	33 (97)	6 (38)
Unknown	2 (5)	1 (3)	10 (63)
**History of ticks**			
Yes	17 (40)	14 (41)	5 (31)
No	23 (55)	18 (53)	0
Unknown	2 (5)	2 (6)	11 (69)

AF fever/anemia group, CC control cat group, SS stray/stable group

Background data from the 91 cats in this study (92 samples): 42 samples were from cats with fever or anemia, 34 were from control cats and 16 cats were from either stables or stray cats. One cat was included twice with two separate episodes with fever

## Results

### Baseline data

Ninety-one cats were included in this study, of which 41 cats were treated at ESAHH for unexplained fever and/or anemia. One cat was included twice, as it had two episodes with fever 22 months apart, resulting in 42 samples from cats with fever and/or anemia. Thirty-four cats were control cats. Sixteen cats were included in group 3, of which four were stray cats trapped in southern Sweden (Scania County) and 12 were stable cats. A summary of these data is available in Table 1. The age in group 3 (stray/stable cat) was considerably lower than in group 1 and 2, but no statistical comparison between the composition of the groups was made.

### Prevalence of antibodies and agents

The estimated prevalence of different antibodies and DNA for the tested agents by group is summarized in Table 2. As there was no sample positive for *Ehrlichia* species DNA, this agent was not included in any statistical analysis.

**Table 2:** Estimated prevalence rates for different pathogens sorted by inclusion group

Summary of test results	Positive test results *n*			
	prevalence % and [confidence interval %]			
Group	AF	CC	SS	Total
Total numbers of samples	42	34	16	92
*Bartonella* spp. IgG	13	17	10	40
	31 [19–46]	50 [34–66]	63 [39–82]	43 [34–54]
*Bartonella* spp. PCR	0	0	1	1
	0 [0.0-8.4]	0 [0.0–10]	6.2 [1.1–28]	1.1 [0.19–5.9]
*Anaplasma/Ehrlichia* species	0	0	0	0
	0 [0.0-8.4]	0 [0.0–10]	0 [0.0–19]	0 [0.0–4.0]
Feline foamy virus antibodies	21	11	10	41
	50 [36–65]	32 [19–49]	63 [39–82]	45 [35–55]
Feline immunodeficiency virus antibodies	0	1	2	3
	0 [0.0-8.4]	2.9 [0.52-15]	13 [3.5–36]	3.3 [1.1–9.2]
Feline leukemia virus antigen	1	0	0	1
	2.4 [0.42-12]	0 [0.0–10]	0 [0.0–19]	1.1 [0.19–5.9]
Felis catus gammaherpesvirus-1 antibodies	25	25	12	62
	60 [45–73]	74 [57–85]	75 [51–90]	67 [57–76]
Haemotropic *Mycoplasma* PCR	2	3	2	7
	4.8 [1.3–16]	8.8 [3.0–23]	13 [3.5–36]	7.6 [3.7–15]
*Toxoplasma gondii* antibodies	15	8	11	34
	36 [23–51]	24 [12–40]	69 [44–86]	37 [28–47]

AF anemia/fever group, CC control cat group, SS stray/stable group

(% positive) [95% Confidence interval]

Prevalence is described as percentage of positive test results, out of tested samples in each category, and 95% confidence interval, as a range of percent

### Associations between agents and background variables

On analysis of background variables, there were significant positive associations between the presence of FFV antibodies and the cat being raised outdoor χ²(df) = [8.7533, 1], *P* = 0.003 and between presence of FFV antibodies and bite wounds during the last 12 months χ²(df) = [4.8312, 1], *P* = 0.028. Post hoc analysis of outdoor access showed significant association between FFV antibodies and outdoor access (but not living strictly outdoor) compared to strictly indoor χ²(df) = [9.5317, 1], *P* = 0.006. There was a significant impact of sex on presence of FFV antibodies (*P* = 0.045). Post hoc analyzes failed to show a significant association between a specific sex category and presence of FFV antibodies. Post hoc analysis of age showed a significant difference between the prevalence of FFV antibodies in cats older than 7 years (58%, CI 36–77%) compared to antibodies in cats younger than 2 years (11%, CI 2.9–31%, *P* = 0.037).

Since there was only one FeLV positive cat, statistical analyses involving this agent were not performed. The FeLV positive cat was a 3-year-old neutered female, with anemia, rectal temperature of 39.4 °C and was housed indoors and evaluated in 2016. The presenting complaints were lethargy, hyporexia, and seizures. The hematocrit at first presentation was 16% which decreased to 13% over the next day. It was treated for immune mediated hemolytic anemia and responded well to prednisolone administration but relapsed whenever treatment was tapered. This cat was negative for hemoplasmas. The cat was negative for FeLV antigen when tested again in 2019 (SNAP FIV/FeLV combo test, IDEXX laboratories, Maine, USA) and was lost to follow up 3 years later.

Cats aged less than 1.75 years of age, had significant lower frequency FcaGHV1 antibodies (35%, CI 17–59%) than either cats aged 1.75–4.5 years (89%, CI 69–97%), χ²(df) = [9.1882, 1], *P* = 0.015 or cats aged 4.5-7 years (86%, CI 65–95%), χ²(df) = [8.2116, 1], *P* = 0.025. Neutered males had higher prevalence of FcaGHV1 antibodies (88%, CI 75–95%) than neutered females (59%, CI 42–74%), χ²(df) = [7.0962, 1], *P* = 0.046. There was a significant positive association between FcaGHV1 antibodies and history of ticks during the last 12 months χ²(df) = [7.15, 1], *P* = 0.007.

The only background variable that showed a significant association with haemotropic *Mycoplasma* spp. was a history of ticks during the last 12 months (*P* = 0.019).

There was a significant association between exposure to *T. gondii* and a history of fleas in the cat (*P* = 0.031) and a history of bite wounds χ²(df) = [4.6464, 1], *P* = 0.031. Whether the cat had outdoor access did result in an initial significant association χ²(df) = [6.7901, 2], *P* = 0.034 with *T. gondii* antibodies, but post hoc analysis failed to find a significant difference between the groups that were strictly indoor (prevalence of antibodies 31%, CI 18–49%), allowed outdoor access (31%, CI 20–46%), or living completely outdoors (67%, CI 42–85%). See Additional file 2.

### Differences in occurrence between inclusion groups

A statistically significant association between inclusion group and findings of FIV antibodies in serum was found (*P* = 0.038). Post hoc analyses did not result in any significant difference at pairwise comparison between inclusion groups. The GLM analysis was not possible to perform for any other background variable than “outdoor access”, which resulted in no significant association.

Pos hoc analyses showed a significant difference χ²(df) = [8.0275, 1], *P* = 0.014 between the *T. gondii* among control cats (23%, CI 12–39%) versus stray/stable cats (69%, CI 44–86%). After generalized linear model analysis, associations between inclusion group and agents ceased with adjustment for the background variables except for sex (*P* = 0.024). Age, “raised indoor or outdoor”, outdoor access, number of additional cats in the household, history of ticks, history of fleas and history of bite wounds are possible confounders for the association between inclusion group and exposure to *T. gondii*. Statistical data is available in Additional file 3 and 4.

### Co-occurrences

Distribution of coinfections by group is summarized in Table 3. There were 22 combinations of infections. The most common coinfection was *Bartonella* and FcGHV1 (13 cats) followed by the combination of *T. gondii*, FcGHV1, and FFV (9 cats). Of the 4 cats with FIV or FeLV, coinfection with FcGHV1 and FFV was detected in all (FeLV, *Bartonella*, FcGHV1 and FFV; FIV, FcGHV1 and FFV; FIV, FcGHV1, FFV and *T. gondii*; and FIV, FcGHV1, FFV, *T. gondii* and hemoplasma, respectively).

**Table 3:** Distribution of positive vector borne agent test results from cats residing in Sweden

Agent combinations	Numbers of cats with the combination of positive tests
All neg	13
B, G	13
T, G, FFV	9
G and FFV	7
T, B, G, FFV	7
G only	6
T, B, G	5
B only	5
FFV alone	3
B, G, FFV	3
B, G, FFV, Hemo	2
T and G	2
T, G, FFV, Hemo	2
T and FFV	2
T and B	2
T only	2
B and FFV	2
T, G, Hemo	1
G, FFV, Hemo	1
FeLV, B, G, FFV	1
FIV, G and FFV	1
FIV, T, G, FFV, Hemo	1
FIV, T, G, FFV	1

T, *Toxoplasma gondii* antibodies; B, *Bartonella* spp. IgG; FeLV, Feline leukemia virus antigen; FFV, Feline foamy virus antibodies; FIV, Feline immunodeficiency virus antibodies; G, Felis catus gammaherpesvirus-1 antibodies; Hemo, Haemotropic *Mycoplasma* PCR

Overall, there were significant positive associations between antibodies to FFV and presence of hemoplasma DNA (*P* = 0.043), between antibodies to FFV and antibodies to *T. gondii* χ²(df) = [6.72, 1], *P* = 0.01 and antibodies to FFV and antibodies to FcGHV1 χ²(df) = [9.16, 1], *P* = 0.003. Statistical data is available in Additional file 5.

## Discussion

In the current study, we estimated the prevalence of multiple agents in cats with or without fever and anemia and stray or stable cats. This is the first report of the prevalence in Sweden for several of the agents.

In the present study, all cats were PCR negative for *A. phagocytophilum*. There are several studies investigating the prevalence of *A. phagocytophilum* in cats in Europe, and at least 18 of them have used PCR. Of those 18 studies, eight studies failed to amplify *A. phagocytophilum* DNA from any cats, and the remaining ten reported a prevalence between 0.3% and 23% [7]. The reports with the highest prevalence were from Milanese stray cats, where 23% of the blood samples contained *A. phagocytophilum* DNA [54], and from southern Portugal where 7.2% of the stray cats were PCR positive compared to only 2.8% of the domestic cats [55]. Our results are in line with the majority of the European studies that reports a prevalence of PCR positive cats of less than 1%, even in studies from areas with a seroprevalence up to 33% [7]. This finding likely relates to short lived rickettsemia after immune responses develop [8].

Failure to amplify DNA of *E. canis. *in any of our samples was expected, due to the lack of a permanent breeding pool of the transmitting tick *Rhipicephalus sanguineus* in Sweden [18].

The cats described here were commonly exposed to *B. henselae* based on an estimated seroprevalence rate of 43%, but DNA was only amplified from one cat. This finding is similar to other studies and likely relates to cats limiting *Bartonella* spp. bacteremia spontaneously over time [22]. A study from Spain reported higher seroprevalence in cats two years old or younger, compared to older cats [56]. The only PCR finding was from a healthy stable cat. Previous studies have found a higher prevalence of antibodies against *Bartonella* species in cats with fever compared to control cats [23] but that DNA from *Bartonella* species was not amplified more often from cats with anemia than control cats [24, 25]. This emphasizes the difficulty in diagnosing illness in an individual cat based on findings of *Bartonella* species [27]. There are more than fifty different reports of prevalence of *Bartonella* species in European cats. The frequency of seropositivity vary between 1% (Lisbon) and 69% (Germany), positive PCR findings vary between 0% (northeast Germany) to 83.5% (Italy) and positive blood culture results range from 0% (Italy) to 53% (France) of the cats [57]. In Sweden, two previous studies have explored the prevalence in cats. One reported a seroprevalence of 25% against *B. elizabethae*, 1% against *B. henselae* and 0% antibodies against *B. quintana* in blood samples from 1998, with regional differences [29]. The highest prevalence was found in the areas close to Stockholm and Gothenburg. The other study reported 3% positive growth - without enrichment - from the blood of healthy cats from the south part of Sweden in 2000 [30].

Previous European studies reported a seroprevalence of FFV in Germany of 39% [58], and in Switzerland of 36% [59], which is slightly lower but not significantly different than the seroprevalence of FFV 45% in the present study. The prevalence of FFV can vary greatly even within a country, as previously shown in studies from the US and Vietnam [52, 60]. In the former, prevalence of FFV ranged from 42 to 75%, and in the latter, a seroprevalence of 29–78% was reported in separate locations. In previous studies from Australia and the US, adult cats had a higher seroprevalence than young animals [52, 61]. In our study, cats older than 7,5 years had significantly higher prevalence of FFV antibodies than cats younger than 1.5 years (58% versus 11%).The effect of sex on the prevalence have varied in different studies [42, 52]. There was no significant association between FFV antibodies and sex in this study.

In the present study there was a significant positive association between having antibodies against FFV and the presence of hemoplasma DNA, antibodies against *T. gondii* and antibodies against FcaGHV1, respectively. This data is not further analyzed for confounding factors or statistically corrected for multiple testing as it was viewed as a purely explorative part of the overall analysis of the data. We might therefore overestimate the positive association between different agents in our study. Our data also showed a significantly increased seroprevalence of FFV in cats with outdoor access, but not in cats that lived strictly outdoors or strictly indoors. The lack of association between cats living strictly outdoors and having FFV antibodies was likely due to the small sample size and possible misclassification of housing status. The prevalence of FFV antibodies was highest in the stray/stable group and these cats were the absolute majority among all cats living strictly outdoors.

A total of three cats (3,3%) were FIV positive in our study. There was a significant impact of inclusion group on the prevalence of FIV and two of the three FIV positive cats were adult, trapped stray cats. Due to the low number of FIV-infected cats, it was not possible to draw any further conclusions in post hoc analyses. However, these results are consistent with previous findings that stray/feral cats are more prone to be FIV infected [62, 63]. The prevalence of FIV vary between different studies. In a Swedish study from 1999, including 96 anesthetized cats, FIV antibodies were not detected in any cat [64]. In a recent Italian study with cats sampled for health checks, the reported prevalence of FIV was 0.8% [65] and a North American study showed a prevalence of FIV of 2.5% of sampled cats [66]. The prevalence of FIV in ill cats in Brazil was 9.8% and 2,2% in healthy cats in a study [67] and another study from Brazil showed an overall prevalence of 5.5% [68]. FIV is notifiable to the Swedish Board of Agriculture. Between 2010 and 2019, 6–42 index cases was reported yearly (median 19.5).

In a recent pan-European study, FeLV RNA was not detected in saliva from 343 cats visiting veterinarians in Sweden [37]. The overall prevalence of FeLV RNA was 2.3% in Europe, and in the previously mentioned Swedish study of 96 anesthetized cats, FeLV antigen was not detected in any cat [49]. In our study, only one cat was seropositive for FeLV. The cat had never traveled abroad. FeLV is notifiable to the Swedish board of Agriculture. Between 2010 and 2019, 6–16 index cases was reported each year (median 8).

Most prevalence studies regarding FcaGHV1 have used results from PCR assays. Reported prevalence of cats with detectable FcaGHV1 DNA in blood samples has varied; ≅ 1% in Italy [69], 1.3% in Japan [70], 6% in Switzerland [71], 9.6% in Singapore [43], 11.4% in Australia [43], 16% in US [42] and 20% in Austria/Germany [44]. The prevalence of antibodies against FcaGHV1 was about twice as high (32%) as the prevalence of FcaGHV1-DNA positive cats (15%) in a study comparing serology and PCR [51]. Our findings, with an average of 67% of the cats having antibodies against FcaGHV1, indicates that exposure to the virus is very common among cats in the southern parts of Sweden, and among all groups in the study, as the variation was small between inclusion groups (61–75%). According to our results, neutered males (88%) were at increased risk for FcaGHV1 exposure compared to neutered females (59%) and age affected the risk. Reported risk factors for being FcaGHV1-DNA positive in other studies were the male sex [43, 44, 71] and age (being adult/older age) in US, Australia [43], Austria [44], Germany [44], Japan [70] and Switzerland [71] and in a closed breeding colony of cats [41]. There is a report from Singapore where the sex distribution of FcaGHV1-positive cats was equal [43]. In a Swiss study, no difference in prevalence between stray and pet cats were reported [71], and in our study, the prevalence was very similar between inclusion groups (61–75%). FIV positive cats are more likely to be FcaGHV1-positive in several reports [43, 44, 70, 71]. There are reports of positive relationship between FcaGHV1 and FeLV infection [43, 71], FcaGHV1 and FIV infection [43, 44, 70, 71] and FcaGHV1 and hemoplasma infection [43, 71, 72]. Our data did not show positive associations between FcCGHV1 and any other agents except FFV, but very few cats were infected by FeLV, FIV or the hemoplasmas.

The total prevalence of hemoplasma DNA in the cats described here was 7.6%. Adequate DNA for sequencing was available from five of seven cats and all were classified as ‘*Candidatus* M. haemominutum’. A recent study from nearby Denmark reported a prevalence of 15% ‘*Candidatus* M. haemominutum’ DNA positive cats and 1.5% *M. haemofelis* DNA positive cats in convenience sampled cats [73]. Other studies have shown a total prevalence of 11–17% and prevalence of 3.3–5.4% for *M. haemofelis* DNA, 0.5–12.6% for ‘*Candidatus*’ M. haemominutum and 6.2% for ‘*Candidatus*’ M. turicensis [74–76]. The numbers of positive cats in our study were too small to detect any differences between inclusion groups.

More than two hundred papers have been published about the seroprevalence of *T. gondii* in cats. A meta-analysis calculated a global seroprevalence of 35% in domestic cats and a seroprevalence of 43% in Europe [77]. This can be compared with an overall seroprevalence of 37% in our study. The lower prevalence (not significant) found in our study, does not necessarily mean that the prevalence of *T. gondii* among cats in in southern Sweden is lower than in Europe in general, or lower today than during the earlier Swedish study published in 1990. The selection of cats sampled will affect the prevalence in different studies. In our study, we found a significant higher seroprevalence in stable and stray cats (69%), compared to control cats (24%), but with several possible confounding factors. Some risk factors previously described for this agent are hunting [78], eating raw meat [79], older age [78–83], outdoor access [78], living in the countryside [78], non-pedigree cat [78, 83, 84], certain cat breeds including Birmans and Ocicats [79], being male [81–84] and originating from a shelter/being stray [81, 82, 85]. We consider the association between bite wounds, fleas and *T. gondii* seropositivity a marker for outdoor access.

The small sample size, especially the low number of positive test results for some of the agents, as well as the non-randomized selection of cats in the control group, requires caution when interpreting the prevalence estimates published here, as they might not be representative of the cat population in the south part of Sweden.

## Conclusions

In the present study, antibodies against FFV and FcaGHV1 were common and prove for the first time that cats in the south part of Sweden are exposed. Antibodies against *Bartonella* spp. were common and suggests that exposure to fleas was frequent. *Candidatus* M. haemominutum’ was the only hemoplasma sequenced from 5 of 7 PCR positive cats suggesting that this may be a commonly detected hemoplasma in the region. As in other studies, each of the agents or antibodies against the agents can be detected in both control cats and ill cats showing that test results alone do not prove disease associations. Overall, we conclude that cats housed indoors (when humanely possible), fed processed foods, and provided flea and tick control will have lower exposure to these agents.

## Electronic supplementary material

Below is the link to the electronic supplementary material.

Supplementary Material 1

Supplementary Material 2

Supplementary Material 3

Supplementary Material 4

Supplementary Material 5

## Data Availability

The datasets used and/or analyzed during the current study are available from the corresponding author on reasonable request.

## Declarations

### Prior publication

The data have not been published previously.

## Abbreviations

ESAHH: Evidensia Specialist Animal Hospital in Helsingborg
FcaGHV1: Felis catus gammaherpesvirus-1
FeLV: Feline leukemia virus
FFV: Feline foamy virus
FIV: Feline immunodeficiency virus
