# Supplementary material for: Prevalence of selected infectious agents in Swedish cats with fever and/or anemia compared to cats without fever and/or anemia and to stable/stray cats
Source: Acta Vet Scand. 2025 May 9;67:23. doi: 10.1186/s13028-025-00807-8 (PMC12063316; doi:10.1186/s13028-025-00807-8)
Supplement: Supplementary file 1 — Supplementary Material 1 [file 13028_2025_807_MOESM1_ESM.docx]

**Additional file 1. Questionnaire for the cat owner (translated from Swedish).**

Date:

Patient label

1. Has your cat been treated with antibiotics in the last 4 weeks?

Yes No

2. Your cat's sex?

Unneutered female Neutered female Unneutered male Neutered male

3. Do you have more cats and if so, how many?

No Yes, _____

4. Where was your cat bred?

Indoors only Fully or partially outdoors

5. Does your cat go outside freely (not on a leash or fenced)?

Never Yes Lives outdoors only

6. Has your cat had ticks in the last 12 months?

Yes No

7. Has your cat fought with another cat in the last 12 months?

Yes No

8. Has your cat had bite wounds in the last 12 months?

Yes No

9. Has your cat had fleas at any time in the last 12 months?

Yes No

10. Has your cat had lice at any time in the last 12 months?

Yes No

11. Has your cat been regularly treated with tick prophylaxis in the last 12 months?

Yes No

What kind?

How often?

12. Has your cat been treated preventively against vermin (fleas, lice) regularly in the last 12 months?

Yes No

With what?

How often?
